# Assuring the quality of diagnostic testing: The future is now

**DOI:** 10.4102/ajlm.v5i2.558

**Published:** 2016-10-17

**Authors:** John Nkengsong, Debrah I. Boeras, Alash’le Abimiku, Rosanna W. Peeling

**Affiliations:** 1 US Centers for Disease Control and Prevention, Atlanta, Georgia, United States; 2 International Laboratory Branch, US Centers for Disease Control and Prevention, Atlanta, Georgia, United States; 3 Institute of Human Virology, University of Maryland School of Medicine, Baltimore, Maryland, United States

In the 1 February 2006 issue of *Clinical Infectious Diseases*, Petti et al.^1^ described laboratory medicine in Africa as a barrier to effective healthcare and highlighted the need for increased investment in laboratory services in Africa. In response to that article, Berkelman et al.^2^ voiced their concern that laboratory services have become the ‘Achilles heel’ of global efforts to combat infectious diseases and antimicrobial resistance.

Now, 10 years later, the future of laboratory medicine in Africa has never been brighter and many have played a part. Countries are committed to using innovative technologies and connectivity solutions to ensure the quality of diagnostic testing, not only in laboratories but at point-of-care (POC) sites across the continent.

The World Health Organization (WHO) has provided leadership through several landmark events, starting with the Maputo Declaration for strengthening laboratory health systems in January 2008,^3^ followed by the Lyon statement on the need for developing countries to establish practical quality management systems in April 2008.^4^ Specifically in Africa, the Yaoundé resolution was issued by the WHO Regional Office for Africa (WHO AFRO) in 2008 to strengthen public health laboratories in the WHO African region.^5^ Since July 2009, several African countries, donors, the WHO and implementing partners launched a programme for strengthening laboratory management, with the aim of accelerating national laboratory services toward ISO 15189 accreditation in the African region. The WHO AFRO accreditation scheme adopts the Stepwise Laboratory Improvement Program Towards Accreditation (SLIPTA) programme. The purpose of SLIPTA is not to replace existing accreditation schemes such as those offered by the College of American Pathologists, International Organization for Standardization, South African National Accreditation System or South African Development Community Accreditation Service, but to serve as a scheme to assist the laboratories in obtaining these internationally-recognised accreditation standards. In 2011, the African Society for Laboratory Medicine (ASLM) was launched as a pan-African professional body working to advocate for the critical role and needs of laboratory medicine and networks throughout Africa as a means of improving the quality of health services. ASLM has become the implementing partner for SLIPTA.^6^

At the same time, another set of driving forces to improve the quality of laboratories and increase access to quality-assured testing came from disease control and elimination programmes for HIV, tuberculosis and malaria. In particular, The President’s Emergency Plan for AIDS Relief, the Bill & Melinda Gates Foundation, UNITAID and others have galvanised industry to develop innovative POC technology platforms. However, implementation of POC tests has been fraught with problems as the health systems in which testing must occur are often weak, fragmented and, experience a critical shortage of healthcare personnel in high-burden countries, ill-prepared for any additional testing. POC testing has now become a double-edged sword in that, while POC tests can increase access to testing and allow patients to be linked to evidenced-based care, its introduction has put tremendous stresses on fragile healthcare systems.

Decentralising testing requires programmes for training and monitoring effectiveness to be amplified a hundred- to a thousand-fold in magnitude, while ensuring quality test results. The introduction of CD4 count testing has shown that managing a network of POC sites has proven to be challenging for Ministries of Health, with significant operator and instrument errors, frequent instrument breakdowns and stock-outs of reagents and other supplies. Technological innovation is not sufficient; innovation in health service delivery is urgently needed.

As part of its UNITAID grant to facilitate the evaluation and implementation of POC tests for CD4, HIV viral load and early infant diagnosis, the London School of Hygiene & Tropical Medicine conducted consultations in nine countries with an interest in quality assurance. These countries have shown commitment and enthusiasm for embracing quality assurance, in particular a system of External Quality Assessment (EQA). As shown in this supplement, these countries have developed policies for ensuring the quality of diagnostics performed at both laboratories and at the POC. Their commitment has not stopped at developing a laboratory or POC policy – the implementation plans include setting up a national system of quality assurance.

To overcome health system constraints, the London School of Hygiene & Tropical Medicine collaborated with the Ministry of Health in Zimbabwe to pilot middleware solutions to show that connectivity allows Ministries of Health to monitor instrument and operator performance across the country. Connectivity solutions can also be used to facilitate improvements in supply chain management to avoid stock-outs and wastage, making health systems more efficient, and to improve patient outcomes.

Quality comes at a cost but, compared with the human and economic costs of not having quality assurance, that cost is insignificant. The London School of Hygiene & Tropical Medicine, in collaboration with the US Centers for Disease Control and Prevention, have developed models and tools for integration of POC and laboratory systems, including costing an overall EQA programme. The model, soon to be posted on the International Diagnostics Centre/London School of Hygiene & Tropical Medicine website, allows countries to estimate and budget for an EQA programme as part of the cost of an overall diagnostic programme. It is now possible to include this cost in the overall cost of introducing a new diagnostic test.

These innovations and tools can be applied to EQA programmes for other diseases. In the last 10 years, WHO AFRO has established networks of laboratories for malaria, tuberculosis, enterics and meningitis in the regions that are linked by EQA programmes. WHO and the US Centers for Disease Control and Prevention published an EQA policy manual for HIV POC testing in 2015. ASLM is committed to sustaining these quality assurance efforts in the region with implementing partners such as the US Centers for Disease Control and Prevention, the Clinton Health Access Initiative and the Elizabeth Glaser Pediatric AIDS Foundation. ASLM as a pan-African organisation can leverage competencies, expertise and the comparative advantage of each organisation, as well as academia to build capacity, mobilise funds and advocate for the value of quality-assured diagnostics to procurement agencies such as UNITAID and the Global Fund.

The outlook for laboratory medicine in the region has never been brighter. The future of diagnostics is now. No new diagnostics should be introduced without first establishing a quality assurance programme and no diagnostic should be procured without including a budget for quality assurance.
