# Corrigendum: Assuring the quality of diagnostic testing: the future is now

**DOI:** 10.4102/ajlm.v6i1.661

**Published:** 2017-07-19

**Authors:** John Nkengasong, Debrah I. Boeras, Alash’le Abimiku, Rosanna W. Peeling

**Affiliations:** 1 US Centers for Disease Control and Prevention, Atlanta, Georgia, United States; 2 International Diagnostic Centre, London School of Hygiene and Tropical Medicine, London, United Kingdom; 3 Institute of Human Virology, University of Maryland School of Medicine, Baltimore, Maryland, United States

In the version of this article initially published, the first author’s surname was misspelled and the affiliations for the second and last authors were misstated. The author list and affiliations are hereby corrected to:

John Nkengasong, US Centers for Disease Control and Prevention, Atlanta, Georgia, United States

Debrah I. Boeras, International Diagnostic Centre, London School of Hygiene and Tropical Medicine, London, United Kingdom

Alash’le Abimiku, Institute of Human Virology, University of Maryland School of Medicine, Baltimore, Maryland, United States

Rosanna W. Peeling, International Diagnostic Centre, London School of Hygiene and Tropical Medicine, London, United Kingdom

The errors have been corrected in the PDF version of the article. We apologise for any inconvenience that this may have caused.

